# The value of lung function assessment and Testin expression detection in clinicopathological features and prognosis of NSCLC patients

**DOI:** 10.1186/s13019-024-02720-z

**Published:** 2024-04-16

**Authors:** Yanmin Zhang, Gaoming Wang, Qian Zhang, Qian Wang, Jing Luo, Chunhua Ling

**Affiliations:** 1 Department of Respiratory and Critical Care Medicine, The First Affiliated Hospital of Soochow University, Suzhou, 215000 Jiangsu P.R. China; 2 Department of Respiratory and Critical Care Medicine, The Second Affiliated Hospital of Xuzhou Medical University, Xuzhou, 221006 Jiangsu P.R. China; 3 Department of Thoracic Surgery, Xuzhou Central Hospital, Xuzhou Clinical School of Xuzhou Medical University, 221006 Jiangsu, P.R. China; 4 Department of Cardiothoracic Surgery, Jinling Hospital, Medical School of Nanjing University, 210000 Nanjing, Jiangsu P.R. China

**Keywords:** NSCLC, Lung function, Testin, Systemic inflammatory response, Prognosis

## Abstract

**Objective:**

The aim of this study is to investigate the clinical value and potential prognostic significance of lung function assessment and Testin expression in non-small cell lung cancer (NSCLC) patients.

**Methods:**

The NSCLC patients were classified into three groups according to lung function: group of normal lung function, group of PRISm (preserved ratio impaired spirometry) (FEV1, forced expiratory volume during the first second < 80% predicted and FEV1/FVC (forced vital capacity) ≥ 70%) and group of COPD (chronic obstructive pulmonary disease) (FEV1/FVC < 70%). The pre-operational clinicopathological characteristics of these patients were recorded and the markers of systemic inflammatory response, including neutrophil to lymphocyte ratio (NLR), lymphocyte to monocyte ratio (LMR), platelet to lymphocyte ratio (PLR) and eosinophils (EOS), were compared between three groups. The expression of Testin in NSCLC samples was detected by IHC and we further explored the correlation between Testin expression and clinicopathological characteristics and prognosis of NSCLC patients. Finally, Cox regression analysis was conducted to study the prognostic factors of NSCLC patients.

**Results:**

Of the 158 NSCLC patients, percentages of normal lung function, PRISm and COPD were 41.4%, 22.8% and 36.1%, respectively. Patients with tumor in the left lung were more likely to have pulmonary dysfunction (PRISm and COPD) than the right lung. The markers of systemic inflammatory response showed differences to various degree in the three groups and NSCLC patients with PRISm or COPD presented more unfavorable prognosis than patients with normal function. The expression of Testin correlated with lymph node metastasis, TNM stage and tumor invasion of NSCLC patients. Moreover, patients with low Testin expression exhibited poorer disease-free survival and overall survival than those with high Testin expression. In Cox regression analysis, we found that PRISm, COPD and Testin expression served as prognostic factors in NSCLC patients.

**Conclusions:**

The presence of COPD or PRISm influenced systemic inflammatory response and prognosis of NSCLC patients. Testin expression correlated with clinicopathological features and could be potentially used as a prognostic marker in NSCLC.

## Introduction

Lung cancer is the dominant cause of cancer-related death worldwide, with NSCLC accounting for nearly 85% [1]. Smoking, airway inflammation and air pollution are the common pathogenic factors of NSCLC [2]. Airway obstruction such as chronic inflammation and even airflow restriction can promote the occurrence and development of NSCLC and affect the response of NSCLC to various treatment [3]. COPD is a major global health problem, which is defined as a FEV1 to FVC ratio smaller than 0.7 [4]. Similar to COPD, PRISm has unique impairment of lung function and do not meet the diagnostic criteria of COPD. As an “non-specific” spirometric pattern, PRISm is defined as a post-bronchodilator FEV1% predicted below 80% with a FEV1/FVC ratio ≥ 0.7 [5]. Both COPD and PRISm have been reported to be associated with prognosis of lung cancer patients [6, 7], but the relationship and molecular links between NSCLC and COPD/PRISm remains not fully elucidated.

Chronic inflammatory and immune responses play vital roles in the development and progression of COPD and PRISm. Serum inflammatory indicators, including neutrophil to lymphocyte ratio (NLR), lymphocyte to monocyte ratio (LMR) and platelet to lymphocyte ratio (PLR) have been reported to predict treatment outcomes and help to identify patients most likely to benefit from treatment especially in immunotherapy [8, 9]. Moreover, previous studies have found a role of blood eosinophils (EOS) in the early response to PD-1 inhibitors in NSCLC patients [10]. In NSCLC patients treated with immune checkpoint inhibitors therapy, eosinophil measurements during treatment might be useful for predicting prolonged treatment failure [11]. However, there is no report on the difference and clinical value of pre-operative peripheral blood NLR/PLR/LMR/EOS in patients with NSCLC under normal lung function compared with PRISm or COPD.

Testin protein (encoded by TES) is expressed in almost all normal human tissues, while low or lack of Testin expression has been found in prostate cancer, endometrial carcinoma, ovarian, breast, acute lymphoblastic leukemia and nasopharyngeal carcinoma [12–17]. As a kind of tumor suppressor, Testin has been widely studied in gastrointestinal tract, breast, head and neck and intracranial tumors, but its research in lung tumors, especially in NSCLC, is less [18]. Our research team previously found that Testin was lowly expressed in NSCLC tissues compared to normal human lung tissues, and ectopic expression of the Testin gene inhibited cancer cell proliferation, invasion and colony formation in NSCLC cells [19]. Up to now, the nature of Testin expression in NSCLC tissues and its clinical significance has not been well clarified.

Herein, we compared the differences of serum inflammatory indicators, prognostic survival and Testin expression in NSCLC patients with PRISm or COPD. Our results indicated that the coexistence of COPD or PRISm influenced systemic inflammatory response and prognosis of NSCLC patients. And Testin expression correlated with clinicopathological characteristics and survival of NSCLC.

## Materials and methods

### Patient selection

**Fig. 1:**
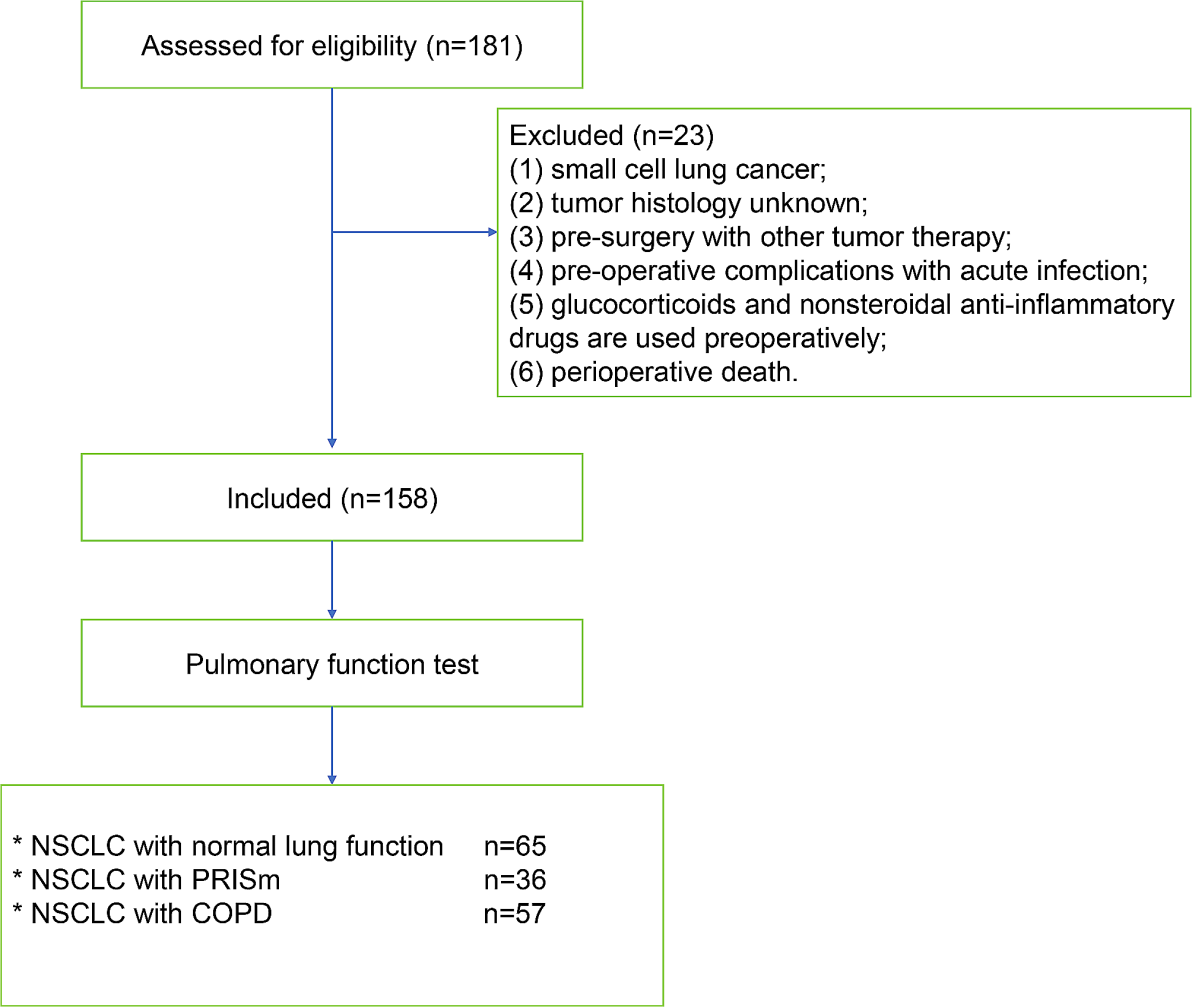
Screening and inclusion process for patients

Lung cancer patients (stages I to IIIA), diagnosed with NSCLC from January 2010 to December 2018 at the Second Affiliated Hospital of Xu Zhou Medical University were consecutively enrolled in the study cohort. Inclusion criteria was: (1) NSCLC was diagnosed pathologically after surgery; (2) patients had undergone a baseline pulmonary function test; (3) complete medical records and follow-up information are available. Exclusion criteria was: (1) small cell lung cancer; (2) tumor histology unknown; (3) pre-surgery with other tumor therapy; (4) pre-operative complications with acute infection; (5) glucocorticoids and nonsteroidal anti-inflammatory drugs are used preoperatively; (6) perioperative death. Totally 158 patients were included in our study. Based on the results of lung function tests, participants were classified into three groups: the normal lung function group included patients with FEV1 ≥ 80% of predicted and FEV1/FVC ≥ 0.7; the PRISm group included patients with FEV1 < 80% of predicted value and FEV1/FVC ≥ 0.7; the COPD group included patients with FEV1/FVC < 0.7 (Fig. 1).

### Data acquisition

By consulting the electronic medical record system, demographic data included age, gender, smoking history, maximum tumor diameter, histological type, lymph node metastasis status, location, invasion, TNM stage (according to the eighth of TNM) classification), lung function and serum inflammatory indicators (NLR, PLR, LMR and EOS). The prognosis data of overall survival (OS) and disease-free survival (DFS) was obtained by phone follow-up and survival intervals were dated at December 31, 2020. In addition, we collected archived formalin-fixed and paraffin-embedded tumor tissues from 158 NSCLC patients. Ki-67 antigen is a protein associated with intracellular division and proliferation, which is expressed in S, G1, G2, and M phases of the cell cycle, and is often used as a reliable marker of tumor cell proliferation activity [20]. The expression of Testin and Ki-67 were analyzed by immunohistochemistry (IHC). Written informed consent was obtained from all patients, and protocols for this study were approved by the Ethics Committee of Second Affiliated Hospital of Xu Zhou Medical University.

### Immunohistochemistry

Tumor Sect. (3-µm) were cut from formalin-fixed paraffin-embedded blocks and mounted on positive-charged slides. The primary antibody was goat anti-Testin and anti-Ki-67 polyclonal antibody (Santa Cruz Biotechnology). The paraffin sections were placed in a xylene bath for 10 min to remove paraffin, and repeated again and then placed in an ethanol gradient for rehydration. Antigen retrieval was performed with EDTA (pH 8.0) repair solution in a microwave, cooled to room temperature, treated with 3% H_2_O_2_ for 10 min for inactivation of endogenous peroxidase, rinsed with 1× PBST (0.1% Tween), incubated with 5% rabbit serum at room temperature for 15 min, and then incubated with primary antibody (1:100) at 4˚C overnight. The sections were then rinsed and incubated with biotin-labeled secondary antibody (SP KIT; Beijing Zhongshan Golden Bridge Biotechnology Co., Ltd., Beijing, China) at 37˚C for 15 min, rinsed in 1× PBST (0.1% Tween) and then incubated with horseradish peroxidase (SP KIT; Beijing Zhongshan Golden Bridge Biotechnology Co., Ltd.) at 37˚C for 15 min. The sections were then treated with DAB for 10 min and the reaction was terminated. All sections were observed in at least five areas at a magnification of 400 by at least two investigators in a blinded manner. Cytoplasm and nuclei were counterstained with hematoxylin solution. Two pathologists who were blinded to patients’ clinical data examined all slides separately. The method of assessing the density of Testin in tumor was as described by our previous studies [19]. In brief, numbers of Testin in tumor were identified according to the immunohistochemical staining and were counted as follows: the total number of cells and positive cells were counted and the staining was scored as the percentages of positive cells: 0 (no staining) for specimens with positive cells ≤ 5%; 1 (weak staining) for specimens with positive cells > 5% and ≤ 25%; 2 (moderate staining) for specimens with positive cells > 25% and ≤ 50%; 3 (strong staining) for specimens with positive cells > 50%. Specimens with scores of ≤ 1 were regarded as negative and specimens with scores of > 1 were regarded as positive.

### Statistical analysis

Continuous variables were presented as medians and inter-quartile ranges (IQRs) and were compared using the Kruskal–Wallis test. Categorical variables, expressed as the numbers and percentages of participants, were compared using the Chi-square test. The survival rate was estimated by using the Kaplan–Meier method and compared using the log-rank (Mantel–Cox) test. Risk factors for survival were analyzed using Cox proportional hazards regression model. Variables with a *p*-value < 0.20 in the univariate analysis were included in the multivariate analysis. In this risk-adjusted analysis, backward stepwise methods were applied to determine the independent factors that were associated with survival. Statistical significance was set at *p* < 0.05. All statistical analyses were performed using the SAS 9.13 software package (version 9.13; SAS Institute, Cary, NC USA) and Prism 8.0.

## Results

### Clinicopathological characteristics of patients

The baseline clinicopathological characteristics of the patients were presented in Table 1. A total of 158 patients were included in our study, among which 91(57.6%) were younger than 65 years old and 123 (77.8%) were male. These patients were divided into NSCLC only (*n* = 65, 41.1%), NSCLC with PRISm (*n* = 36, 22.8%) and NSCLC with COPD (*n* = 57, 36.1%) groups based on pulmonary function. Clinicopathological characteristics of the patients were compared, and results indicated that there were no statistically significant differences in age, gender, smoking history, maximum tumor diameter, histological type, lymph node metastasis status, invasion, Testin expression, Ki-67 expression among the three groups (*p* > 0.05). Whereas the three groups differed in tumor location. Patients with tumor in the left lung were more likely to develop pulmonary dysfunction (PRISm and COPD) than the right lung (*p* = 0.027).

**Table 1:** Clinicopathological parameters of included patients

Characteristics	Overall	NSCLS only	NSCLC with PRISm	NSCLC with COPD	*P* value
	*n* = 158	*n* = 65(41.1%)	*n* = 36(22.8%)	*n* = 57(36.1%)	
**Age(years)**					**0.428**
≤ 65	91(57.6%)	41(63.1%)	18(50%)	32(56.1%)	
>65	67(42.4%)	24(36.9%)	18(50%)	25(43.9%)	
**Gender**					**0.237**
Male	123(77.8%)	54(83.1%)	28(77.8%)	40(70.2%)	
Female	35(22.2%)	11(16.9%)	8(22.2%)	17(29.8%)	
**Smoking status(pack/year)**					**0.464**
<30	69(43.7%)	28(43.1%)	13(36.1%)	28(49.1%)	
≥ 30	89(56.3%)	37(56.9%	23(63.9%)	29(50.9%)	
**Maximum tumor diameter (cm)**					**0.672**
≤ 3	60(37.95%)	27(41.5%)	12(33.3%)	21(36.8%)	
3< d ≤ 5	60(37.95%)	21(32.3%)	14(38.9%)	25(43.9%)	
5< d ≤ 7	20(12.7%)	7(10.8%)	6(16.7%)	7(12.3%)	
>7	18(11.4%)	10(15.4%)	4(11.1%)	4(7%)	
**Histological type**					**0.421**
Squamous	77(48.7%)	30(46.2%)	21(58.3%)	26(45.6%)	
Adenocarcinoma	66(41.8%)	26(40%)	13(36.1%)	27(47.4%)	
Others	15(9.5%)	9(13.8%	2(5.6%)	4(7%)	
**pN status**					**0.228**
N0	89(56.3%)	32(49.2%)	26(72.2%)	31(54.4%)	
N1	34(21.5%)	15(23.1%)	6(16.7%)	13(22.8%)	
N2-N3	35(22.2%)	18(27.7%)	4(11.1%)	13(22.8%)	
**Location**					**0.027**
Right lung	76(48.1%)	39(60%)	12(33.3%)	25(43.9%)	
Left lung	82(51.9%)	26(40%)	24(66.7%)	32(56.1%)	
**Invasion**					**0.455**
Yes	71(44.9%)	30(46.2%)	13(36.1%)	28(49.1%)	
NO	87(55.1%)	35(53.8%)	23(63.9%)	29(50.9%)	
**Textin expression**					**0.943**
High	86(54.4%)	36(55.4%)	20(55.6%)	30(52.6%)	
Low	72(45.6%)	29(44.6%)	16(44.4%)	27(47.4%)	
**Ki-67**					**0.415**
≤ 30	63(39.9%)	27(41.5%)	11(30.6%)	25(43.9%)	
>30	95(60.1%)	38(58.5%)	25(69.4%)	32(56.1%)	

### Serum inflammatory indicators of the three groups

**Fig. 2:**
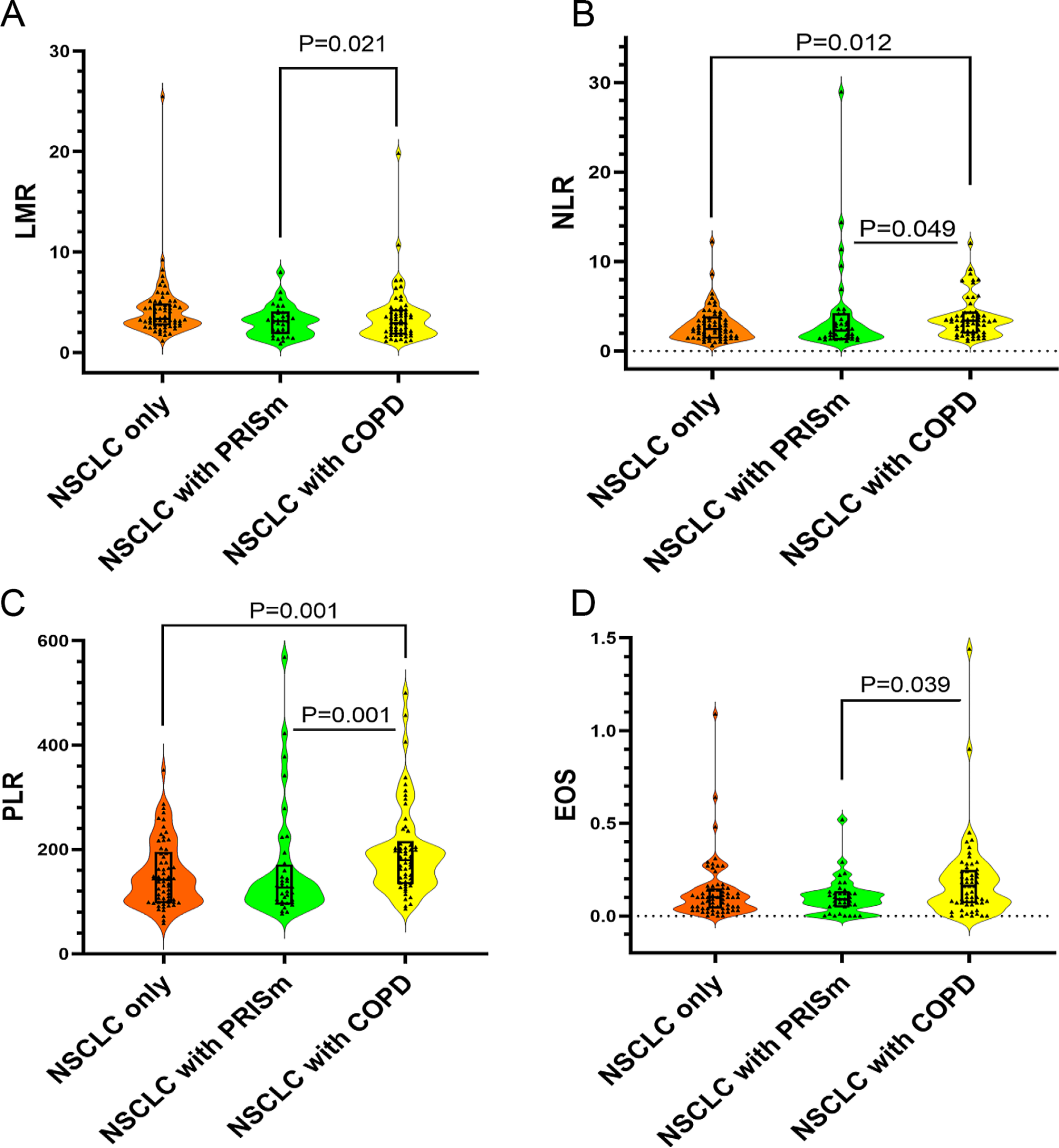
Characteristics of serum inflammatory indicators in three groups. (**A**) Lymphocyte to monocyte ratio (LMR) in three groups. (**B**) Neutrophil to lymphocyte ratio (NLR) in three groups. (**C**) Platelet to lymphocyte ratio (PLR) in three groups. (**D**) Eosinophils (EOS) in three groups

Lymphocyte to monocyte ratio (LMR) of COPD group was higher than that of PRISm group (*p* = 0.021) (Fig. 2A**)**. For neutrophil to lymphocyte ratio (NLR), it was higher in COPD group compared with NSCLC only group (*p* = 0.012) or PRISm group (*p* = 0.049) (Fig. 2B**)**. Similarly, platelet to lymphocyte ratio (PLR) was higher in COPD group compared with NSCLC only group (*p* = 0.001) or PRISm group (*p* = 0.001) (Fig. 2C**)**. As for eosinophils (EOS), we found that it was higher in COPD group compared with PRISm group (Fig. 2D**)**.

### Prognosis of the three groups

**Fig. 3:**
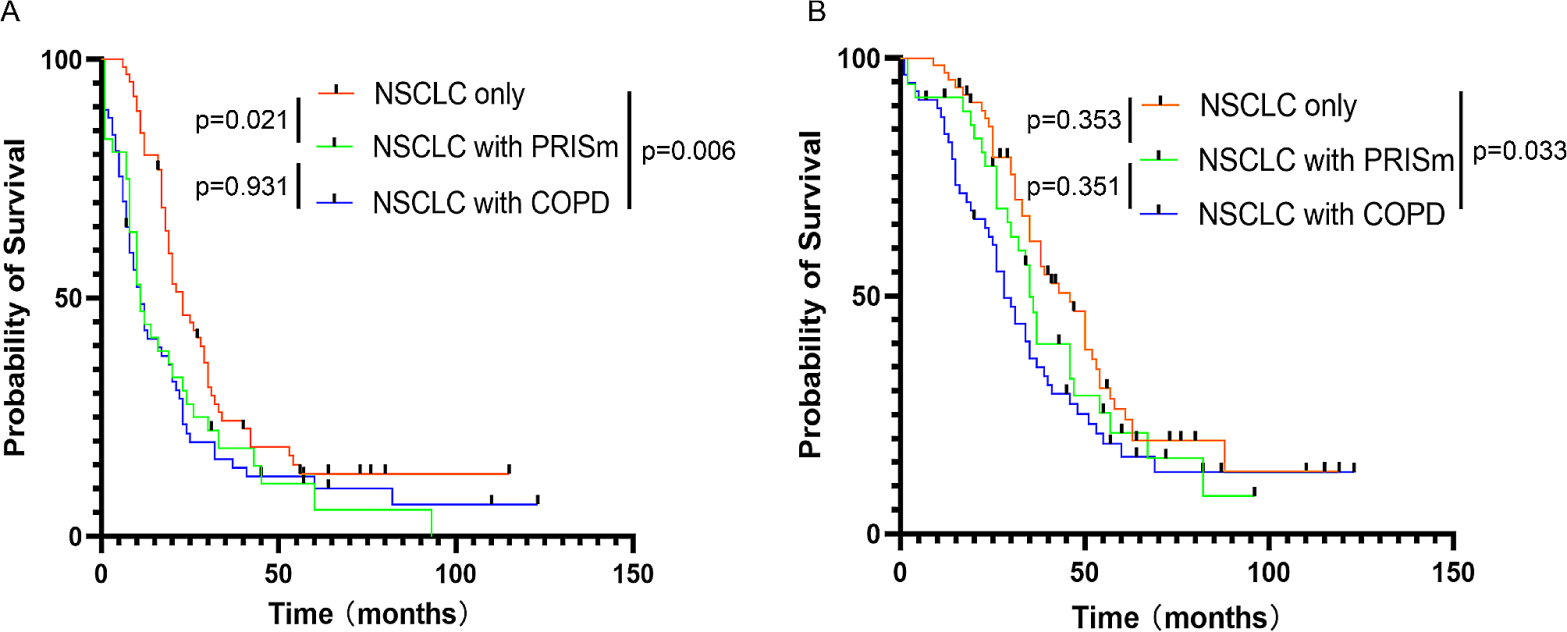
Prognosis of the three groups. (**A**) Disease-free survival in three groups. (**B**) Overall survival in three groups

There were significant differences of disease-free survival (DFS) in PRISm (*p* = 0.021) and COPD (*p* = 0.006) group compared with NSCLC only group, while there was no difference between PRISm and COPD group (*p* = 0.931) (Fig. 3A**)**. For overall survival (OS), there was significant difference between NSCLC only group and COPD group (*p* = 0.033). Nevertheless, no statistic difference of overall survival was found between other groups (NSCLC only group vs. PRISm group, *p* = 0.353; PRISm group vs. COPD group, *p* = 0.351) (Fig. 3B).

### Testin expression with clinicopathological characteristics of NSCLC patients

**Fig. 4:**
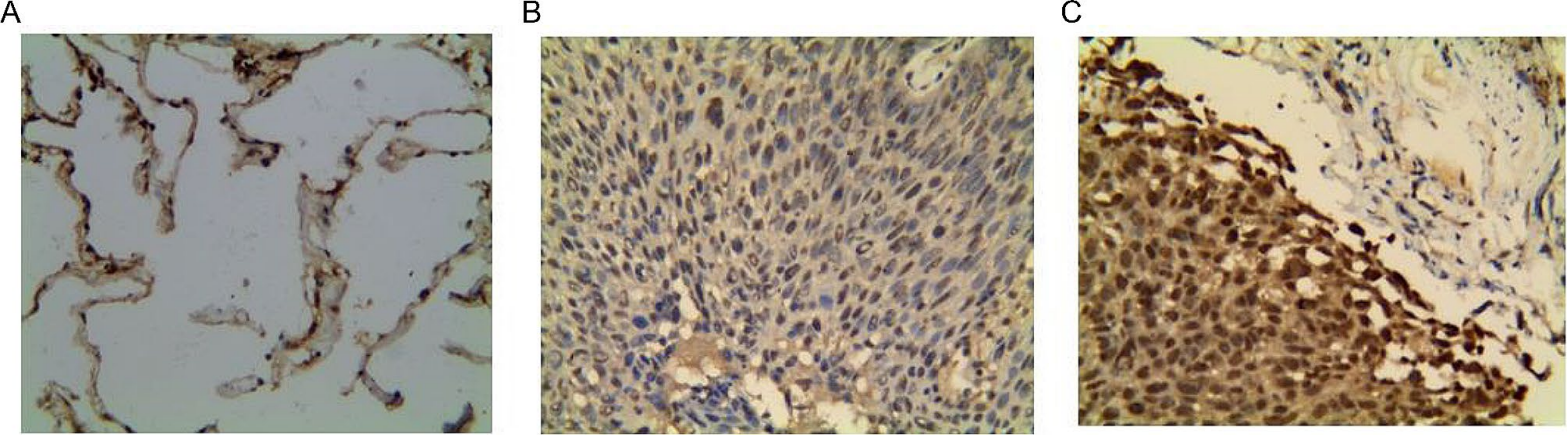
Examples of Testin in NSCLC. (**A**) No Testin expression. (**B**) Testin low expression. (**C**) Testin high expression

The expression of Testin protein in 158 NSCLC patients was detected by IHC (Figure 4A-C). These patients were divided into group of Testin low expression and group of Testin high expression according to the IHC staining of Testin. Combined with the clinicopathological characteristics of patients, we found that Testin expression correlated with pN status (*p* = 0.006), TNM stage (*p* = 0.0012) and tumor invasion (*p* = 0.0055). Patients of Testin low expression exhibited more advanced characteristics in lymph node metastasis, TNM stage and tumor invasion (Table 2).

**Table 2:** Clinicopathological characteristics of patients grouped by Testin expression

Clinicopathological features	Testin		Total	*P* Value
	Low (*n* = 72)	High(*n* = 86)		
**Age (years)**				**0.425**
≤ 65	39(54.2%)	52(60.5%)	91(57.6%)	
>65	33(45.8%)	34(39.5%)	67((42.4%)	
**Gender**				**0.129**
Male	60(83.3%)	63(73.3%)	123(77.8%)	
Female	12(16.7%0	23(26.7%)	35(22.2%)	
**Maximum tumor diameter (cm)**				**0.494**
≤ 3	25(34.7%)	35(40.7%)	60(37.95%)	
3 < d ≤ 5	26(36.1%)	34(39.5%)	60(37.95%)	
5 < d ≤ 7	10(13.9%)	10(11.6%)	20(12.7%)	
> 7	11(15.3%)	7(8.2%)	18(11.4%)	
**Histological type**				**0.580**
Squamous	32(44.4%)	45(52.3%)	77(48.7%)	
Adenocarcinoma	32(44.4%)	34(39.5%)	66(41.8%)	
others	8(11.2%)	7(8.2%)	15(9.5%)	
**Tumor differentiation**				**0.642**
Low-medium	42(58.3%)	47(54.7%)	89(56.3%)	
Medium-high	30(41.7%)	39(45.3%)	69(43.7%)	
**pN status**				**0.006**
N0	29(40.3%)	60(69.8%)	89(56.3%)	
N1	19(26.4%)	15(17.4%)	34((21.5%)	
N2-N3	24(33.3%)	11(12.8%)	35(22.2%)	
**Ki-67**				**0.162**
≤30	33(45.8%)	30(34.9%)	63(39.9%)	
>30	39(54.2%)	56(65.1%)	95(60.1%)	
**TNM stage**				**0.0012**
I	8(9.4%)	24(32.9%)	32(20.3%)	
II	36(42.4%)	22(30.1%)	58(36.7%)	
III	41(48.2%)	27(37.0%)	68(43.0%)	
**Invasion**				**0.0055**
Yes	41(56.9)	30(34.9)	71(44.9%)	
NO	31(43.1)	56(65.1)	87(55.1%)	
**Group**				**0.472**
NSCLC only	26(36.1%)	39(45.3%)	65(41.1%)	
PRISm	17(23.6%)	19(22.1%)	36(22.8%)	
COPD	29(40.3%)	28(32.6%)	57(36.1%)	

### Testin expression with prognosis of NSCLC patients

**Fig. 5:**
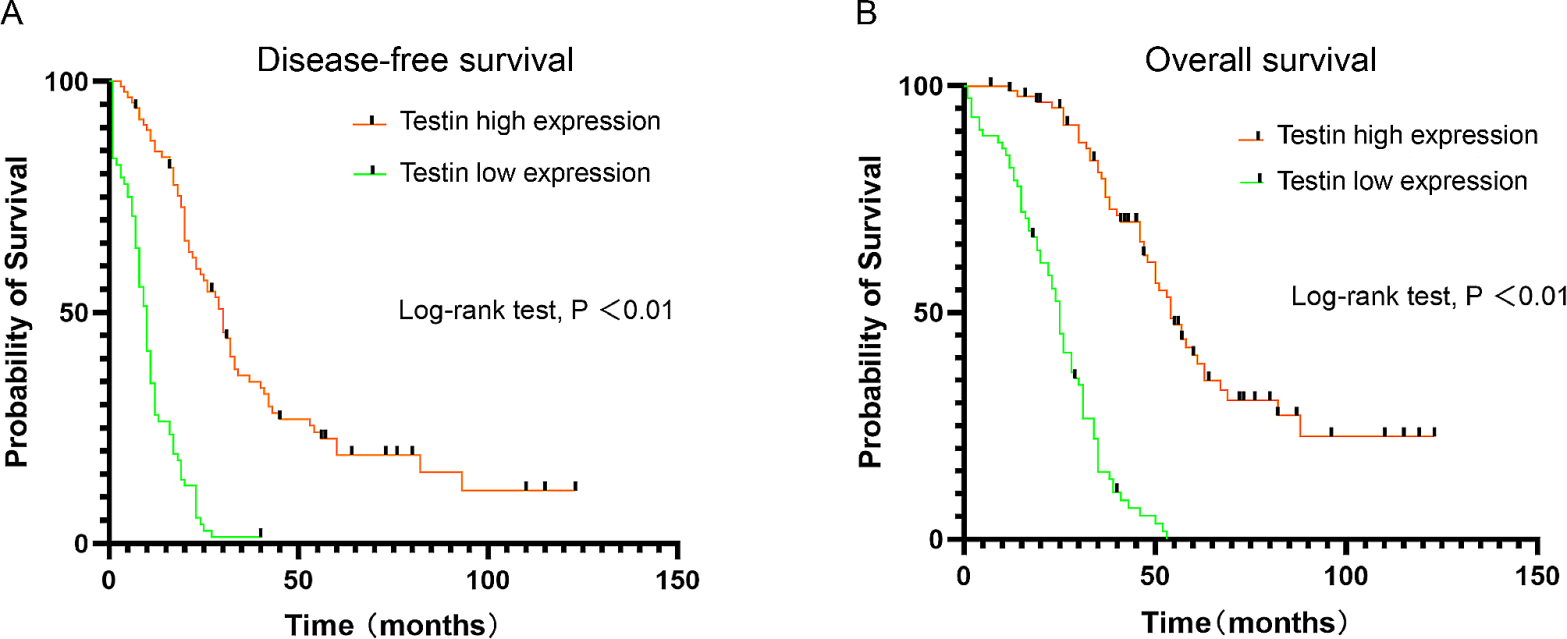
The relationship between Testin expression and prognosis. (**A**) Testin expression with disease-free survival; (**B**) Testin expression with overall survival

Patients with Testin low expression had poorer disease-free survival(10.0 ± 10.0 months)than those with Testin high expression(27.5 ± 26.0 months) (*p*<0.01) (Fig. 5A**)**. Likewise, patients with Testin low expression had more unfavorable overall survival time(25.0 ± 16.75 months) than those with Testin high expression(47.0 ± 27.5 months)(*p*<0.01) (Fig. 5B).

### Prognostic analysis of NSCLC patients

Univariate Cox regression was performed to analyze the prognostic factors of NSCLC. Results showed that old age (*p* = 0.0294), stage III (*p* = 0.0079), Testin low expression (*p* < 0.0001), N1-N3 (*p* = 0.0053), low-medium differentiation (*p* = 0.0438), invasion (*p* < 0.0001), group of PRISm or COPD (*p* = 0.0082) were associated with poor prognosis for disease-free survival (DFS). While old age (*p* = 0.0002), stage III (*p* = 0.0002), Testin low expression (*p* < 0.0001), T3-T4 (*p* = 0.0042), N1-N3 (*p* = 0.001), invasion (*p* = 0.0055), group of PRISm or COPD (*p* = 0.0264) were associated with poor prognosis for overall survival (OS) (Table 3). Furthermore, multivariate Cox regression analysis revealed that age (HR = 1.041; 95%CI = 1.021–1.064; *p* < 0.0001), Testin expression (HR = 0.156; 95%CI = 0.101–0.241; *p* < 0.0001), Ki-67 expression (HR = 1.624; 95%CI = 1.120–2.354; *p* = 0.0106), invasion (HR = 1.866; 95%CI = 1.281–2.720; *p* = 0.0012) and group of PRISm or COPD (HR = 1.291; 95%CI = 1.056–1.577; *p* = 0.0126) were independent prognostic factors for disease free survival (DFS) (Table 4). And for overall survival (OS), age (HR = 1.067; 95%CI = 1.043–1.092; *p* < 0.0001), stage (HR = 1.584; 95%CI = 1.075–2.334; *p* = 0.0200), Testin expression (HR = 0.123; 95%CI = 0.076–0.198; *p* < 0.0001) and group of PRISm or COPD (HR = 1.299; 95%CI = 1.048–1.610; *p* = 0.017) were independent prognostic factors (Table 5).

**Table 3:** Univariate Cox regression

Variables	Disease free survival			Overall survival		
	Hazard radio	95% CI	p	Hazard radio	95% CI	p
**Gender**			**0.382**			**0.4896**
Male	1			1		
Female	1.194	0.803–1.775		1.165	0.757–1.795	
**Age**			**0.0294**			**0.0002**
≤ 60	1			1		
> 60	1.022	1.002–1.043		1.042	1.020–1.066	
**Stage**			**0.0079**			**0.0002**
I-II	1			1		
III	1.59	1.129–2.239		2.026	1.404–2.923	
**Testin expression**			**<0.0001**			**<0.0001**
Low	1			1		
High	0.193	0.130–0.287		0.141	0.090–0.219	
**Ki-67**			**0.2764**			**0.8062**
≤ 30	1			1		
> 30	1.209	0.859-1.700		1.047	0.725–1.513	
**pT status**			**0.0548**			**0.0042**
T1-T2	1			1		
T3-T4	1.4	0.993–1.975		1.707	1.183–2.464	
**pN status**			**0.0053**			**0.001**
N0	1			1		
N1-N3	1.62	1.154–2.272		1.844	1.280–2.658	
**Pathology**			**0.3625**			**0.0559**
S	1			1		
A-AS Mixed	1.169	0.835–1.636		1.428	0.991–2.058	
**Differentiation**			**0.0438**			**0.0953**
Low-medium	1			1		
Medium-high	0.707	0.505–0.990		0.734	0.511–1.056	
**Invasion**			**<0.0001**			**0.0055**
None	1			1		
Yes	2.022	1.421–2.877		1.714	1.171–2.507	
**Tumor size (cm)**			**0.2377**			**0.2328**
≤ 5	1			1		
>5	1.267	0.855–1.876		1.288	0.850–1.950	
**Group**			**0.0082**			**0.0264**
NSCLC only	1			1		
PRISm or COPD	1.29	1.068–1.558		1.262	1.028–1.551	

**Table 4:** Multivariate Cox regression of disease free survival

Variables	Disease free survival		
	Hazard radio	95% CI	p
Age	1.042	1.021–1.064	<0.0001
Testin	0.156	0.101–0.241	<0.0001
Invasion	1.866	1.281–2.720	0.0012
Group of PRISm or COPD	1.291	1.056–1.577	0.0126

**Table 5:** Multivariate Cox regression of overall survival

Variables	Overall survival		
	Hazard radio	95% CI	p
Age	1.067	1.043–1.092	<0.0001
Stage	1.584	1.075–2.334	0.02
Testin	0.123	0.076–0.198	<0.0001
Group of PRISm and COPD	1.299	1.048–1.610	0.017

## Discussion

Chronic airway inflammation plays an important role in the occurrence and development of chronic obstructive pulmonary disease and lung cancer [21]. Siemes et al. followed up a group of more than 7000 people without malignant tumors for about 10 years. The results showed that the possibility of cancer increased when CRP (C-reactive protein) > 3 mg/dl [22]. Danish researchers measured baseline CRP, fibrinogen and leukocyte levels in 8656 patients with COPD and followed them up for 5 years [23]. It was found that patients with elevated inflammatory indexes had a four-fold increased risk of lung cancer. PRISm was previously considered to be an unclassified and restricted lung function impairment disease [24]. Like COPD, it has the change of decreased FEV1 and chronic airway inflammation. Patients with PRISm are more likely to be male, smoker, and tend to have a higher basis of metabolic syndrome and cardiovascular and cerebrovascular diseases [25, 26].A recent study from South Korea demonstrated that NSCLC patients with PRISm had a worse survival prognosis than those with COPD or normal lung function [6]. And COPD was associated with postoperative development of respiratory failure after thoracic surgery [27]. Moreover, for patients with COPD, smoking cessation is useful to improve symptoms, respiratory function and metabolic parameters in the short term [28]. In our study, we found that patients with PRISm or COPD exhibited poorer disease-free survival (DFS) than the normal lung function group. When comparing the overall survival (OS) of NSCLC patients with different lung function states in the three groups, there was only significant difference between COPD group and normal lung function group. The reason why it is not completely consistent with previous studies may be related to the baseline level differences of the patients, such as the selected population, sample size, age and tumor stage.

In the occurrence, development and prognosis of chronic lung inflammation and lung tumors, systemic inflammatory indicators, such as NLR, LMR, PLR and EOS, can play a critical role in innate and adaptive immunity as effective markers [29, 30]. Neutrophils are constantly recruited and activated in COPD lungs, producing a large number of oxidants such as ROS, causing DNA oxidative damage and increasing the incidence of lung tumors. Neutrophil elastase secreted by neutrophils can directly activate TLR4 signaling pathway, thus promoting the expression of CXCL8 in bronchial epithelial cells and the production of mucin by activating EGFR [31, 32]. From the perspective of adaptive immunity, COPD pulmonary inflammation affects the ability of CD8^+^ cells to clear tumor cells, and impacts the balance of Th1/Th2, the proportion of regulatory T cells and Th17 cells, and the expression of programmed cell death protein 1 (PD-1) and its ligand (PD-L1) in CD8^+^ T cells, resulting in cell cycle arrest and T cell inactivity [33]. A study including 3,656 patients showed NLR to be potentially a useful biomarker to predict the poor prognosis for NSCLC [34]. The research of *Lin* et al. indicated that LMR was potentially used as an independent prognostic factor for survival in previously untreated metastatic NSCLC [35]. In a study comprising 3430 patients, *Gu* et al. found that PLR was associated with unfavorable survival in advanced NSCLC [36]. Similarly, peripheral EOS was reported to be connected with the effect of immune checkpoint inhibitor treatment in NSCLC patients [37]. In our study, we found that there were differences to various degree of serum inflammatory indicators in different lung function groups. The coexistence of PRISm or COPD influenced the systemic inflammatory response of NSCLC patients and these results provided a basis for the individualized treatment and management of NSCLC patients with pulmonary dysfunction.

Testin is a protein expressed in a wide range of normal human tissues and locates in the cytoplasm along stress fibers being recruited to focal adhesions [38]. Latest reports indicated that Testin served as a tumor suppressor in the carcinogenesis of multiple types of cancers, including colorectal cancer [39], childhood acute lymphoblastic leukaemia [40],nasopharyngeal carcinoma [17], breast cancer [41] and gastric cancer [42], however the mechanism of loss of Testin expression is still unknown. Testin participates in the processes of cell cycle, tumor growth, apoptosis, epithelial-mesenchymal transition, angiogenesis, and metastasis [18], suggesting its potential usage in the diagnosis and therapy of cancer. Our previous study identified that Testin expression was reduced in NSCLC cell lines and overexpression of Testin significantly inhibited tumor growth of NSCLC both in vitro and in vivo [19]. In the present study, our results revealed that although there was no difference in Testin expression among the three groups of different pulmonary function, Testin expression correlated with poor clinicopathological parameters of NSCLC patients, including pN status, TNM stage and tumor invasion. Furthermore, patients with relative higher expression of Testin survive longer than those with lower ones and Cox regression analysis indicated that Testin served as an independent risk factor of prognosis for NSCLC patients. Our study adds the understanding of Testin in NSCLC and provides potential markers for the diagnosis and treatment of NSCLC.

In conclusion, this study assessed the pre-operational clinicopathological characteristics of NSCLC patients with PRISm or COPD. NSCLC patients with different lung function exerted differences in systemic inflammatory response and prognosis. Testin was clinically relevant with NSCLC and served as a promising marker for predicting prognosis of operatable NSCLC patients.

## Data Availability

Not applicable.
